# Antimalarial Activity of Orally Administered Curcumin Incorporated in Eudragit^®^-Containing Liposomes

**DOI:** 10.3390/ijms19051361

**Published:** 2018-05-04

**Authors:** Elisabet Martí Coma-Cros, Arnau Biosca, Elena Lantero, Maria Letizia Manca, Carla Caddeo, Lucía Gutiérrez, Miriam Ramírez, Livia Neves Borgheti-Cardoso, Maria Manconi, Xavier Fernàndez-Busquets

**Affiliations:** 1 Nanomalaria Group, Institute for Bioengineering of Catalonia (IBEC), The Barcelona Institute of Science and Technology, Baldiri Reixac 10-12, ES-08028 Barcelona, Spain; elisabet.marti@isglobal.org (E.M.C.-C.); abiosca@ibecbarcelona.eu (A.B.); elantero@ibecbarcelona.eu (E.L.); lucia.gutierrez@isglobal.org (L.G.); miriam.ramirez@isglobal.org (M.R.); lborgheti@ibecbarcelona.eu (L.N.B.-C.); 2 Barcelona Institute for Global Health (ISGlobal, Hospital Clínic-Universitat de Barcelona), Rosselló 149-153, ES-08036 Barcelona, Spain; 3 Nanoscience and Nanotechnology Institute (IN2UB), University of Barcelona, Martí i Franquès 1, ES-08028 Barcelona, Spain; 4 Department of Scienze della Vita e dell’Ambiente, Sezione di Scienze del Farmaco, University of Cagliari, Via Ospedale 72, 09124 Cagliari, Italy; mlmanca@unica.it (M.L.M.); caddeoc@unica.it (C.C.); manconi@unica.it (M.M.)

**Keywords:** malaria, curcumin, nanomedicine, oral administration, lipid nanovesicles, Eudragit, Nutriose, hyaluronan, *Plasmodium yoelii*

## Abstract

Curcumin is an antimalarial compound easy to obtain and inexpensive, having shown little toxicity across a diverse population. However, the clinical use of this interesting polyphenol has been hampered by its poor oral absorption, extremely low aqueous solubility and rapid metabolism. In this study, we have used the anionic copolymer Eudragit^®^ S100 to assemble liposomes incorporating curcumin and containing either hyaluronan (Eudragit-hyaluronan liposomes) or the water-soluble dextrin Nutriose^®^ FM06 (Eudragit-nutriosomes). Upon oral administration of the rehydrated freeze-dried nanosystems administered at 25/75 mg curcumin·kg^−1^·day^−1^, only Eudragit-nutriosomes improved the in vivo antimalarial activity of curcumin in a dose-dependent manner, by enhancing the survival of all *Plasmodium yoelii*-infected mice up to 11/11 days, as compared to 6/7 days upon administration of an equal dose of the free compound. On the other hand, animals treated with curcumin incorporated in Eudragit-hyaluronan liposomes did not live longer than the controls, a result consistent with the lower stability of this formulation after reconstitution. Polymer-lipid nanovesicles hold promise for their development into systems for the oral delivery of curcumin-based antimalarial therapies.

## 1. Introduction

**Figure 1:**
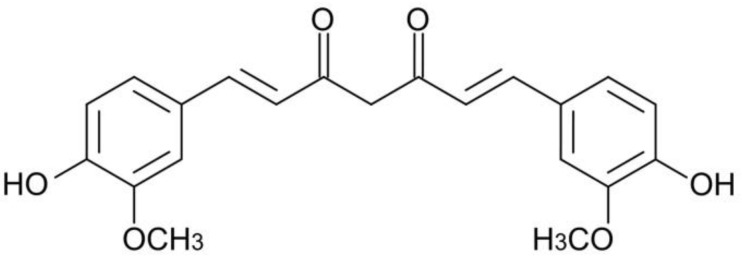
Chemical structure of curcumin.

The polyphenolic compound curcumin (Figure 1) has been described to inhibit the in vitro growth of chloroquine-resistant *Plasmodium falciparum* in a dose-dependent manner with a half-maximal inhibitory concentration (IC_50_) between 5–18 µM [1,2]. In vivo, oral administration of curcumin to mice infected with the murine malaria parasite *Plasmodium berghei* reduced blood parasitemia by 80% to 90% and significantly enhanced survival. Curcumin was able to delay the death of animals by about 10 days and prevent cerebral malaria, the most severe and rapidly fatal neurological complication of the disease [3,4,5,6]. In addition to having a direct antiplasmodial effect, curcumin has also been described to prime the immune system against *P. berghei* [4]. In order to expand this potential as antimalarial, curcumin derivatives have been synthesized and some of them demonstrated an improved in vitro IC_50_ around 400 nM [7]. This inexpensive molecule is easily isolated from rhizome extracts of turmeric (*Curcuma longa*) and has shown low toxicity in animals [8] and humans [9], even when used at high doses: as much as 8 g/day administered for three months to cancer patients on trial did not exhibit toxic side effects [10]. Despite these promising results, the clinical use of curcumin is often limited by its extremely reduced aqueous solubility and high tissue distribution [11]. Low serum and tissue levels of curcumin are observed regardless of the route of administration due to extensive intestinal and hepatic metabolism along with rapid elimination, which contribute to restrain its bioavailability [12,13,14,15]. When administered orally, curcumin undergoes conjugation leading to the formation of curcumin glucuronide and sulphates in the intestinal wall [16], which together with its strong interaction with bile salts contribute to reduce its absorption [17]. In addition, curcumin is a highly unstable molecule, sensitive to various conditions like light, pH and temperature [18,19].

Attempts to overcome these shortcomings of curcumin have contemplated its incorporation in different types of nanoparticles [20,21,22,23,24]. The oral administration of drugs in nanocarriers represents an attractive strategy because it can ensure local delivery and accumulation in the intestines, thus improving systemic bioavailability [25,26,27]. Binding of curcumin to chitosan nanoparticles increased its chemical stability and when fed to mice in this nanoparticulate formulation, it significantly prolonged animal survival when compared to the free compound [28]. While treatment of infected mice at low parasitemia (less than 2%) with curcumin-bound chitosan nanoparticles resulted in survival of all the animals, if the treatment were started at higher parasite loads survival was not 100% [28]. Among the different nanosystems, liposomes have been widely employed due to their safety and versatility. The incorporation of curcumin in phospholipid vesicles can modulate its interaction with the gut mucosa but these formulations cannot ensure drug stability in the gastrointestinal environment due to the phospholipid degradation under such conditions [29,30]. Electrostatic interactions with external ions play an important role in the stability of liposomes, as they can cause an alteration of the structure and size, with possible rupture and content leakage [31,32]. To overcome this limitation, the appropriate combination of phospholipids with gastro-resistant polymers and/or fibres can offer protection to the vesicles, resulting in an enhanced local and systemic bioavailability of the payload. Phospholipid vesicles have been developed incorporating different natural polymers, such as chitosan, sodium alginate, xanthan gum, gelatin, pectin and cellulose ethers [31,33,34]. Liposomes were also coated with the methacrylic acid copolymer Eudragit^®^ S100 to improve vesicle stability [35], and curcumin-Eudragit solid dispersions [25] have been proposed as treatment for gastric ulcer [36] and for colon-specific drug delivery [37]. Through an environmentally friendly preparation method previously employed to produce hyalurosomes [38], Eudragit was combined with hyaluronan to protect liposomes from gastric pH and promote curcumin deposition in the intestines [39]. Liposomes prepared without the polymers were not suitable for pharmaceutical applications, as they were large in size (diameter ≥ 700 nm), polydispersed (polydispersity index ≥ 0.70) and unstable, displaying curcumin precipitation in a short time. Alternatively, new phospholipid nanovesicles designed for intestinal delivery were developed by simultaneously loading the water-soluble dextrin Nutriose^®^ FM06 and curcumin. These innovative vesicles, termed nutriosomes, improved the local and systemic bioavailability and the biodistribution of curcumin upon oral administration [40]. Of note, both Eudragit-hyaluronan liposomes and nutriosomes were freeze-dried to preserve the carrier’s performance and improve their stability on storage, which usually represents one of the major limitations to commercial applications.

In the present work, we combined Eudragit with hyaluronan or Nutriose and phospholipids, thus producing Eudragit-hyaluronan liposomes and Eudragit-nutriosomes, respectively, to protect curcumin from degradation and ameliorate its efficacy. Because these properties are highly desirable for the currently required oral administration of drugs in non-complicated malaria, we have explored the capacity of these two nanovesicles to improve the antiplasmodial activity of curcumin in an in vivo model of the disease.

## 2. Results

### 2.1. Nanovesicle Characterization

Eudragit-hyaluronan liposomes and Eudragit-nutriosomes were prepared according to the compositions reported in Table 1.

The mean diameter of freshly prepared (before freeze-drying) Eudragit-hyaluronan liposomes and Eudragit-nutriosomes was approximately 1100 nm and the polydispersity index (PI) was >0.6 (Table 2). After freeze-drying, the samples were re-hydrated with water and sonicated, resulting in the production of much smaller vesicles with narrower size distribution (~150 nm and PI < 0.3). The zeta potential was not affected by the freeze-drying process: values were around −30 mV for both formulations. Eudragit-hyaluronan liposomes and Eudragit-nutriosomes showed the same values of entrapment efficiency (79.3 ± 5.6% and 81 ± 8.7%, respectively).

**Figure 2:**
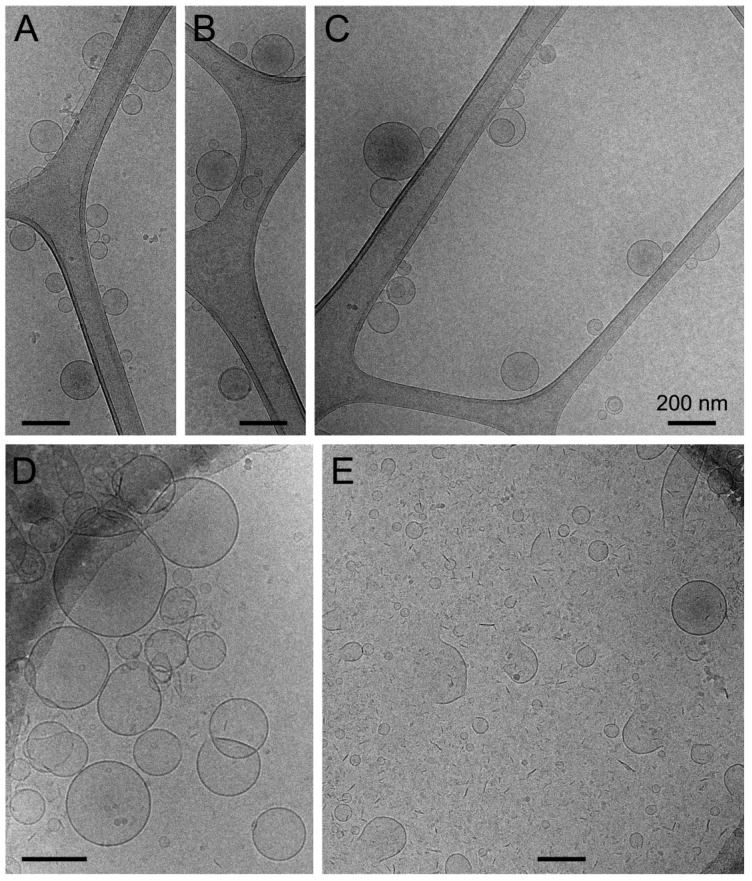
Cryo-TEM analysis of rehydrated curcumin-incorporating (**A**–**C**) Eudragit-nutriosomes and (**D**,**E**) Eudragit-hyaluronan liposomes. Scale bars represent 200 nm.

Cryo-transmission electron microscopy (cryo-TEM) analysis revealed for rehydrated Eudragit-nutriosomes incorporating curcumin the presence of well-formed vesicles with the expected size around 150 nm in diameter [40] (Figure 2A–C). On the other hand, curcumin-containing Eudragit-hyaluronan liposomes were slightly larger in size (Figure 2D), but their cryo-TEM images revealed the presence of abundant disassembled vesicles and membrane fragments, suggesting a certain instability of these structures (Figure 2E).

### 2.2. Vesicle Behavior in Gastrointestinal Fluids

Since pH and ionic strength variations along the gastrointestinal tract can affect vesicle performance, the behaviour of Eudragit-hyaluronan liposomes and Eudragit-nutriosomes under conditions mimicking gastric and intestinal media was evaluated. To this aim, two high ionic strength solutions at pH 1.2 and pH 7.0 were assayed. After 2 h at acidic pH, an increase in size and polydispersity was observed for Eudragit-hyaluronan liposomes (>200 nm, PI > 0.3; Table 3), while Eudragit-nutriosomes showed no sign of physical alteration, as demonstrated by the fact that their size was essentially unchanged (~150 nm, PI ~ 0.2). An inversion of the zeta potential to around +13 mV was detected for both formulations, due to the presence of H^+^ in the acidic medium. Similar results were observed after 6 h of incubation at pH 7.0, confirming that Eudragit-nutriosomes were more stable than Eudragit-hyaluronan liposomes, as the structure of the former was preserved despite pH variation and ionic strength.

### 2.3. In Vivo Antimalarial Activity of Orally Administered Curcumin

**Figure 3:**
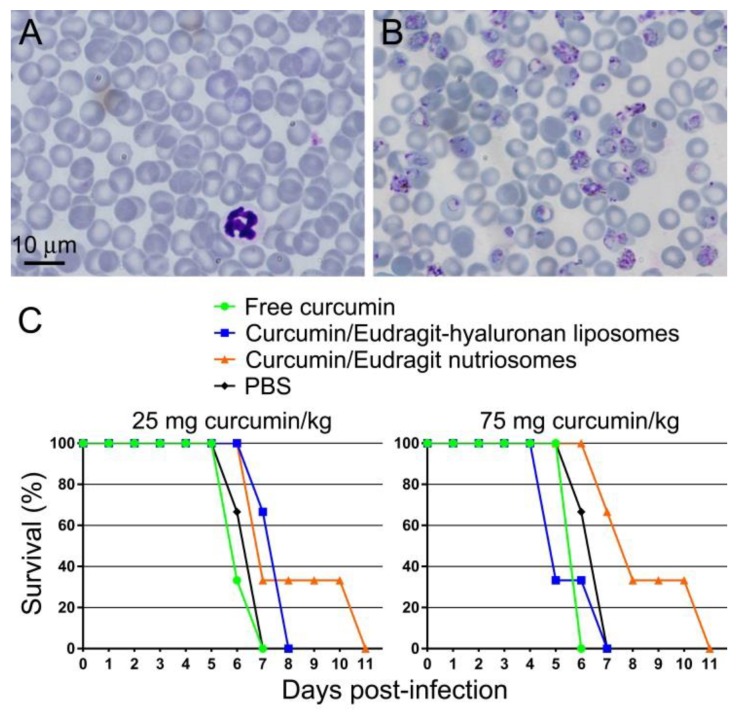
In vivo antimalarial activity of orally administered curcumin. Representative microscope images of blood smears from (**A**) non-infected and (**B**) *P. yoelii*-infected mice. (**C**) Kaplan-Meier plot for the in vivo assay of the effect on *P. yoelii*-infected mice (*n* = 3 animals/sample) of free or incorporated curcumin administered orally at 25 and 75 mg·kg^−1^·day^−1^.

Free curcumin administered orally at either 25 or 75 mg·kg^−1^·day^−1^ to mice infected with the lethal murine malaria parasite *Plasmodium yoelii yoelii* 17XL did not have a curative effect on the animals, which died at day 6 or 7 after infection, similarly to untreated controls (Figure 3). The same amount of curcumin incorporated in Eudragit-hyaluronan liposomes did not improve significantly this result. On the other hand, identically administered curcumin incorporated in Eudragit-nutriosomes was able to increase mouse viability in a dose-dependent manner. At both 25 and 75 mg·kg^−1^·day^−1^, all Eudragit-nutriosome-curcumin-treated mice lived longer than free curcumin controls, with two animals surviving until day 11 post-infection. Because polyphenol entrapment efficiency in the nanoformulated preparations was about 80%, the performance of the nanocarriers is underestimated due to the presence in these samples of about 20% of non-incorporated curcumin.

**Figure 4:**
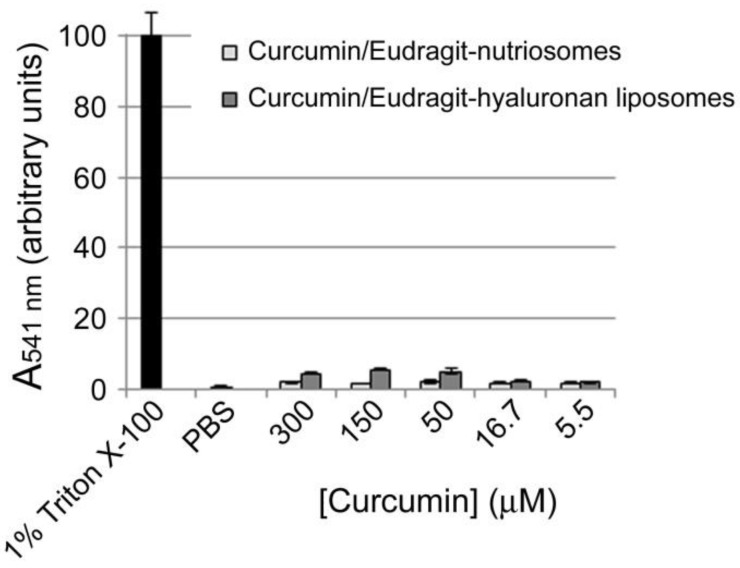
Hemolytic activity of rehydrated curcumin-incorporating Eudragit-nutriosomes and Eudragit-hyaluronan liposomes. The 300 µM curcumin sample contained 2.3 or 3.1 mg/mL of Eudragit-hyaluronan liposomes or Eudragit-nutriosomes, respectively.

Assuming a homogeneous distribution of the administered formulations throughout mouse tissues, the highest concentration assayed did not exhibit hemolytic activity (Figure 4).

## 3. Discussion

Because of their biocompatibility and physicochemical diversity, lipids offer vast opportunities in nanosystems for oral drug delivery [41]. When applied for the administration of poorly water-soluble drugs, liposomes enhance drug solubilization by creating a lipophilic environment and acting as a shelter that protects incorporated sensitive active ingredients from direct interaction with the aggressive gastrointestinal tract fluids. However, maximum advantages from lipid nanocarriers can only be obtained if they survive the transit through the acidic gastric environment. The dilution in the gastric fluid and the enzymatic breakdown of lipids may result in changes in the composition and structure of the nanocarriers, which leads to the unwanted burst drug release [42]. In comparison with lipid-based nanosystems, polymeric nanovessels are generally stable during gastrointestinal tract transit [43], and advances in polymer chemistry enable exquisite control over their nanoarchitecture and biophysical properties, which facilitates controlled drug delivery [44]. In order to address the multifaceted oral delivery challenges, polymer-lipid systems have been developed with the aim of combining the valuable features of both polymeric and lipid-based structures [33].

In a previous report, Eudragit and hyaluronan were combined with phospholipids to obtain Eudragit-hyaluronan liposomes loaded with curcumin [39]. The association of Eudragit and hyaluronan had a dramatic impact on the vesicles, facilitating their assembly and stability, along with curcumin loading. The polymers, by interacting with each other and with the phospholipid, were supposed to produce a network in which both the vesicles and curcumin were embedded and stabilized, with a consequent improvement of the loading and retention of the polyphenol. Further, the polymers favoured both the re-arrangement of vesicles upon rehydration of the freeze-dried formulation with water and the re-dispersion of curcumin. The vesicles’ integrity was preserved from the gastric environment and the local accumulation of curcumin in the intestines was promoted. In another study, curcumin was incorporated in a novel vesicular carrier system, nutriosomes, made of phospholipid and a water-soluble dextrin (Nutriose) [40], an association that improved vesicle features, stability and performances in vitro and in vivo. Nutriose localized both in the inter-lamellar and inter-vesicle media, leading to a stabilization of the vesicle structure. The dextrin acted also as a cryo-protector, avoiding vesicle collapse during freeze-drying and as a protector against high ionic strength and pH changes encountered in the gastrointestinal environment.

Although the combination of Eudragit with hyaluronan within phospholipid vesicles had been described to increase their stability and promote curcumin deposition in the intestines, it was not able to improve the systemic absorption of the polyphenol [39]. In agreement with this, we did not observe an improvement of the in vivo antimalarial activity of curcumin loaded in Eudragit-hyaluronan liposomes relative to the free compound. However, the important role of other glycosaminoglycans (GAGs) related to hyaluronan as receptors of *Plasmodium*-infected red blood cells (for a review see [45]) suggests that this type of GAG-containing nanovectors can find relevant applications in treatments for some of the most lethal pathological complications of malaria, such as sequestration in the placenta and in the brain capillaries. As an example, the GAG chondroitin sulphate has been described as one of the receptors in the placental sequestration of *P. falciparum*-infected red blood cells [46,47] and in their cytoadherence to vascular endothelial cells lining the small blood vessels of the brain, leading to their blockade in cerebral malaria [48,49]. A study reporting on the effect of polyethylene glycol functionalization of nanoparticles on their intestinal transepithelial transport concluded that anionic polymers are transported primarily through the paracellular route [50]. Given the anionic nature of GAGs, their use in polymer-lipid vesicles holds promise for future orally administered treatments against severe malaria.

We have shown that curcumin incorporated in Eudragit-nutriosomes significantly improved its antimalarial activity upon oral administration to *P. yoelii*-infected mice. The superior performance of Eudragit-nutriosomes versus Eudragit-hyaluronan liposomes could be reasonably explained by the greater stability of the former, as indicated by the vesicle behaviour in gastrointestinal fluid studies. A previous report also demonstrated that nutriosomes composed of P90G (160 mg/mL), curcumin (10 mg/mL) and Nutriose (50 mg/mL) enhanced the accumulation of curcumin in the intestines and the passage of the polyphenol through the intestinal mucosa, increasing its systemic bioavailability [40].

Curcumin has been proposed as an attractive partner for artemisinin, the main currently available drug used to treat severe falciparum malaria. However, artemisinin plus curcumin liposomal formulations have been only assayed for parenteral delivery [51], being oral intake the preferred form of administration of antimalarial treatments for non-complicated malaria due to the poor basic medical infrastructure of many endemic regions. This socioeconomic landscape of malaria, where distribution networks for medicines often become disrupted, makes highly attractive a drug delivery system like the one described here, which can be transported and stored in lyophilized form until its use. Our results indicate that polymer-lipid vesicles are worth exploring for the future development of orally administered curcumin-containing combination therapies against malaria.

## 4. Materials and Methods

### 4.1. Materials

Soy phosphatidylcholine (Phospholipon^®^ 90G, P90G) was purchased from Lipoid GmbH (Ludwigshafen, Germany). Low molecular weight hyaluronan (200–400 kDa) was purchased from DSM Nutritional Products AG Branch Pentapharm (Aesch, Switzerland). Eudragit^®^ S100 with molecular weight about 125 kDa was provided by Evonik Industries AG (Darmstadt, Germany). Nutriose^®^ FM06, a soluble dextrin from maize, was kindly provided by Roquette (Lestrem, France). Curcumin, ethanol and other reagents of analytical grade were purchased from Sigma-Aldrich (Milan, Italy).

### 4.2. Sample Preparation

The following curcumin-polymer dispersions were prepared: (A) curcumin (100 mg) and hyaluronan (50 mg) in 10 mL of bidistilled water; (B) curcumin (100 mg) and Eudragit^®^ S100 (500 mg) in 10 mL of ethanol; (C) curcumin (100 mg) and Nutriose^®^ FM06 (1000 mg) in 10 mL of bidistilled water. All dispersions were stirred for 2 h at 25 °C. To produce Eudragit-hyaluronan liposomes, dispersion A (1.5 mL) was mixed with dispersion B (0.5 mL), and 360 mg of P90G was added and sonicated (45 cycles, 5 s on/2 s off, 13 µm probe amplitude) with a high intensity ultrasonic disintegrator (Soniprep 150, MSE Crowley, London, UK). To produce Eudragit nutriosomes, dispersion B (0.5 mL) was mixed with dispersion C (1.5 mL); thereafter 360 mg of P90G was added and sonicated as above. The resulting vesicle dispersions were frozen at −80 °C and freeze-dried for 24 h at −90 °C in a FDU-8606 freeze-dryer (Operon, Gimpo, Korea). Each sample was rehydrated with 2 mL of water to achieve a curcumin concentration of 10 mg/mL and sonicated (30 cycles, 5 s on/2 s off) prior to use. The composition of the two formulations is reported in Table 1.

### 4.3. Vesicle Characterization

Vesicle formation and morphology were evaluated by cryo-TEM analysis. A thin film of each sample was formed on a holey carbon grid and vitrified by plunging (kept at 100% humidity and room temperature) into ethane maintained at its melting point, using a Vitrobot (FEI Company, Eindhoven, The Netherlands). The vitreous films were transferred to a Tecnai F20 TEM (FEI Company) and the samples were observed in a low dose mode. Images were acquired at 200 kV at a temperature ~−173 °C, using low-dose imaging conditions with a CCD Eagle camera (FEI Company). The average diameter, polydispersity index and zeta potential of vesicles were determined by dynamic and electrophoretic light scattering using a Zetasizer nano-ZS equipment (Malvern Instruments, Worcestershire, UK) [52]. Prior to analysis, the samples were diluted with water (1:500). Non-incorporated curcumin was removed by dialysis of 1 mL of rehydrated sample against water (2.5 L) using Spectra/Por^®^ membranes (12–14 kDa MW cut-off, 3 nm pore size; Spectrum Laboratories Inc., DG Breda, The Netherlands) at 25 °C for 2 h, replacing water every 30 min. The amount of curcumin in each formulation was determined after disruption of the vesicles with methanol (1:1000) by measuring A_424 nm_ with a UV/Vis spectrophotometer (Lambda 25, Perkin Elmer, Waltham, MA, USA). Entrapment efficiency (%) is defined as the amount of curcumin found in dialysed formulations versus that found in non-dialysed formulations. Hemolysis assays were done as described elsewhere [53].

### 4.4. Vesicle Behavior in Gastrointestinal Fluids

The average diameter, polydispersity index and zeta potential of Eudragit-hyaluronan liposomes and Eudragit-nutriosomes were measured after dilution (1:500 *v*:*v*) at 25 and 37 °C and incubation at pH 1.2 for 2 h and at pH 7.0 for 6 h, at 37 °C, in the presence of 0.3 M NaCl, which was used to increase the ionic strength in the media.

### 4.5. In Vivo Antimalarial Activity Assay

The in vivo antimalarial activity of free and nanoformulated curcumin was analysed in a 4-day blood suppressive test as described previously [54]. Briefly, BALB/c mice were inoculated 2 × 10^7^ red blood cells from *P. yoelii yoelii* 17XL-infected mice by intraperitoneal injection. Treatment started 4 h later (day 0) with a single dose of 25 or 75 mg·kg^−1^·day^−1^ curcumin administered (in free form or incorporated in vesicles) by a 150-μL oral delivery followed by identical dose administration for the next 3 days. The samples were prepared at appropriate doses in phosphate-buffered saline (PBS) and the control groups received PBS. Parasitemia was monitored daily by microscopic examination of Giemsa-stained thin blood smears and antimalarial activity was calculated from day 3 by using the Plasmoscore 1.3 software (Burnet Institute, Melbourne, Australia).

## Figures and Tables

**Table 1:** Composition (expressed as mg/mL of water in rehydrated samples) of Eudragit-hyaluronan liposomes and Eudragit-nutriosomes.

Nanovesicles	P90G	Curcumin	Hyaluronan	Eudragit	Nutriose
Eudragit-hyaluronan liposomes	180	10	3.75	12.5	–
Eudragit-nutriosomes	180	10	–	12.5	75

**Table 2:** Mean diameter (MD), polydispersity index (PI) and zeta potential (ZP) of curcumin-loaded Eudragit-hyaluronan liposomes and Eudragit-nutriosomes. Each value represents the mean ± standard deviation, *n* ≥ 3.

Treatment	Nanovesicles	MD (nm)	PI	ZP (mV)
Before freeze-drying	Eudragit-hyaluronan liposomes	1113 ± 109	0.75 ± 0.09	−39.9 ± 2.5
	Eudragit-nutriosomes	1141 ± 96	0.61 ± 0.11	−35.8 ± 3.1
After freeze-drying and sonication	Eudragit-hyaluronan liposomes	156 ± 12	0.29 ± 0.03	−36.2 ± 2.6
	Eudragit-nutriosomes	151 ± 8	0.24 ± 0.05	−33.7 ± 3.7

**Table 3:** Mean diameter (MD), polydispersity index (PI) and zeta potential (ZP) of rehydrated curcumin-loaded Eudragit-hyaluronan liposomes and Eudragit-nutriosomes diluted and incubated at 37 °C in acidic solution (pH 1.2) for 2 h or in neutral solution (pH 7.0) for 6 h, both containing 0.3 M NaCl. The measurements were carried out immediately after dilution (t_0_) at 25 and 37 °C, and after 2 (t_2_) or 6 h (t_6_) of incubation at 37 °C. Mean values of ≥3 replicates ± SD are reported.

pH	Nanovesicles	Time, Temperature	MD (nm)	PI	ZP (mV)
pH 1.2	Eudragit-hyaluronan liposomes	t_0_, 25 °C	138 ± 12	0.32 ± 0.05	+12.5 ± 0.4
		t_0_, 37 °C	144 ± 28	0.40 ± 0.09	+14.0 ± 0.6
		t_2_, 37 °C	215 ± 26	0.35 ± 0.09	+12.6 ± 0.9
	Eudragit-nutriosomes	t_0_, 25 °C	144 ± 10	0.21 ± 0.03	+14.1 ± 0.6
		t_0_, 37 °C	145 ± 8	0.22 ± 0.08	+14.7 ± 0.7
		t_2_, 37 °C	142 ± 8	0.21 ± 0.06	+13.8 ± 0.6
pH 7.0	Eudragit-hyaluronan liposomes	t_0_, 25 °C	167 ± 6	0.27 ± 0.04	−6.0 ± 5.2
		t_0_, 37 °C	226 ± 11	0.26 ± 0.02	−6.0 ± 4.5
		t_6_, 37 °C	193 ± 8	0.23 ± 0.01	−6.2 ± 3.6
	Eudragit-nutriosomes	t_0_, 25 °C	148 ± 4	0.22 ± 0.02	−2.4 ± 0.3
		t_0_, 37 °C	155 ± 7	0.23 ± 0.05	−1.1 ± 0.5
		t_6_, 37 °C	160 ± 4	0.23 ± 0.04	−3.0 ± 0.7
